# Genome-Wide Identification of NAC Gene Family and Expression Analysis under Abiotic Stresses in *Avena sativa*

**DOI:** 10.3390/genes14061186

**Published:** 2023-05-29

**Authors:** Lei Ling, Mingjing Li, Naiyu Chen, Xinying Xie, Zihui Han, Guoling Ren, Yajie Yin, Huixin Jiang

**Affiliations:** Heilongjiang Provincial Key Laboratory of Oilfield Applied Chemistry and Technology, College of Bioengineering, Daqing Normal University, Daqing 163712, China; 13644602460@163.com (L.L.);

**Keywords:** *Avena sativa*, *NAC* transcription factor, evolutionary analysis: gene expression

## Abstract

In this study, a total of 177 *NAC* members were identified in *Avena sativa*, located on 21 chromosomes. Phylogenetic analysis showed that AsNAC proteins could be divided into seven subfamilies (I–VII), and that proteins in the same subfamily have similar protein motifs. Gene structure analysis found that *NAC* introns ranged from 1 to 17. Cis-element analysis of the promoter indicated that the gene family may have stress-related elements and growth regulation elements. Through qRT-PCR experiments, we speculated that *AsNACs* genes can respond to abiotic stresses such as cold, freezing, salt, and saline alkali. This study provides a theoretical basis for further exploring the function of the *NAC* gene family in *A. sativa.*

## 1. Introduction

NAC transcription factors are commonly found in plants. The NAC domain, which is located in the N-terminal region (NAC domain) of these NAC proteins, is extremely conserved (about 160 amino acids) and participates in DNA binding. On the other hand, the C-terminal region is highly variable in both length and sequence and is considered a transcription-activating domain [1]. Additionally, the N-terminal domain of the NAC gene is divided into A–E subdomains [2]. Subdomains C and D have DNA-binding properties, subdomain A may be beneficial to both homodimerization and heterodimerization, whereas subdomains B and E have various functions in NAC TFs. These genes influence plant growth, enhance the absorption of mineral elements, and improve crop nutrition and quality. The NAC TFs are involved in mediating a variety of physiological activities in plants [3,4].

NAC transcription factors are extensively involved in plant growth, abiotic stress, and hormone signal transduction. Earlier studies have shown that NAC TFs play an important regulatory role in plants subjected to abiotic stress including salinity, drought, cold, or abscisic acid (ABA) [5]. RD26/ANAC072 plays a significant role in hormone signaling and is involved in abscisic acid (ABA), methyl jasmonate (JA), and salicylic acid (SA)-induced abiotic stress [6]. Over expression of OsNAC6 can activate the expression of peroxidase-related genes in transgenic rice plants and enhance rice tolerance to high salt stress [7]. Over-expressed grape VaNAC26 can enhance antioxidant capacity and drought and salt tolerance by controlling the expression of stress-related genes and JA synthesis in transgenic *Arabidopsis thaliana* [8]. In addition, the OsNAP gene of the NAC transcription factor family can prevent water loss under abiotic stress by stimulating ABA-mediated stomatal closure, improving drought resistance and salt tolerance of rice [9].

*A. sativa* is an annual herbaceous crop belonging to the family Gramineae and exists in diploid, tetraploid, and hexaploid forms. *A. sativa* is an economic grass family of Poaceae. Cultivated *A. sativa* exists as an allohexaploid (AACCDD, 2n  =  6x  =  42), and *A. sativa* has the potential to replace animal-based foods because of its low carbon footprint and great health benefits. Recently, the genome of cultivated hexaploid *A. sativa* has been published [10]. It is a globally cultivated crop that provides significant economic benefits, but its yield tends to decline under abiotic stress. Abiotic stress causes a significant decrease in cell water and chlorophyll content, which in turn affects the plant’s ability to obtain sufficient substances from photosynthesis [11]. With the development of whole genome sequencing, several NAC members have been studied in model plants. NAC genes have been identified in more and more species, such as Arabidopsis, rice, and wheat [1,12,13], but the NAC family members have not been systematically analyzed and studied in *A. sativa*. In this study we conduct a comprehensive biological analysis of NAC gene in *A. sativa*, including phylogenetic relationship, conserved domain, gene structure, promoter cis-element, and qRT-PCR analysis. We also predicted the gene function of NAC, providing a theoretical reference for further study of the function of NAC transcription factors in the future.

## 2. Materials & Methods

### 2.1. Identification of NAC Genes

Based on the genome data of *A. sativa*, we download the annotated genes and proteins from the database (https://wheat.pw.usda.gov/GG3/node/922, accessed on 3 March 2021) (Table S1). We used two methods (BLAST search and hidden Markov model (HMM)) to identify the NAC genes. BLAST searches with already known *Arabidopsis thaliana* and *Oryza sativa* NAC sequences identified cations of the candidate NAC genes in *A. sativa*. The integrity of the NAC motif was determined by the online SMART (http://smart.embl-heidelberg.de, accessed on 6 March 2021) with an e-value ≤ 0.01 [14]. In addition, we predicted the basic information of the NAC protein, including length, isoelectric point, and molecular weight, using the online ExPASy program (http://www.expasy.org/tools, accessed on 5 April 2021) [15].

### 2.2. Phylogenetic Analysis of NAC Genes

We performed sequence alignments by using ClustalX to investigate the phylogenetic relationship of the NAC gene family in oat [16]. We used the MEGA 7.0 program software to build the neighbor-joining (NJ) phylogenetic tree [17]. Genes were classified according to the homology for *Arabidopsis thaliala* and *O*. *sativa*.

### 2.3. Motif Analysis, Gene Structures, and Conserved Domains of NAC Genes

Jalview software (http://www.jalview.org, accessed on 14 April 2021 ) was used to carry out multiple protein sequence alignment. We used the MEME software (http://meme.ebi.edu.au/meme/intro.html, accessed on 16 April 2021) to identify the conserved motifs and domains, and the maximum number of motifs was set to 15. A gene structure displaying server program (http://gsds.cbi.pku.edu.cn/index.php, accessed on 5 May 2021) was used to show the NAC gene’s structures. 

### 2.4. Gene Duplication and Collinearity Analysis of NAC Genes

We mapped the physical locations of the NAC genes on chromosomes by using Circos (http://circos.ca, accessed on 13 May 2021). The analysis of synteny among the *A. sativa*, *A. thaliana*, and *O. sativa* genomes was conducted locally using a similar way to the one developed for the PGDD (http://chibba.agtec.uga.edu/duplication, accessed on 25 May 2021) [18]. BLAST and OrthoMCL software (http://orthomcl.org/orthomcl/about.do#release, accessed on 10 June 2021) were used to find out the potential homologous gene pairs across various genomes [19,20]. These homologous pairs were used as inputs on the PGDD database (http://chibba.agtec.uga.edu/duplication/, accessed on 18 June 2021) [18].

### 2.5. Cis-Elements Analysis of NAC Genes

To understand the NAC gene family, we analyzed the cis-elements of NAC promoters. We detected sequences within 1500 (bp) upstream of initiation codons (ATG) for promoter analysis and looked for these sequences in the oat genome. The cis-elements were searched for in promoters using the PlantCARE database (http://bioinformatics.psb.ugent.be/webtools/plantcare/html, accessed on 30 June 2021).

### 2.6. Gene Regulatory Network Analysis of NAC Protein

We used BLAST to compare *A. thaliana* and *O. sativa*, selected the protein sequences of the NAC transcription factor in the *A. sativa* genome database, and localized them in the *A. thaliana* Information Resource Database to determine the protein sequences of *A. thaliana* NAC.

Interactions between NAC and other proteins were predicted using the PAIR website (http://www.cls.zju.edu.cn/pair, accessed on 21 July 2021) to map the network of interactions in Cytoscape 3.0.

### 2.7. Synthesis of cDNA, RNA Extraction, and Real-Time PCR Fluorescence Quantification

Total RNA was extracted from whole seedlings by using an RNA extraction kit (DP430 TianGen Biotech, Beijing, China). According to the manufacturer’s protocol, the RNA quality was evaluated using 1.0% (*w*/*v*) agarose gel stained with ethidium bromide (EB), followed by a DNase I treatment to remove DNA contaminations (Takara, Shiga-ken, Japan). The cDNA was synthesized using the Transcriptor first strand cdna synthesis Kit (Indianapolis, IN, USA) and used as a template for gene expression analysis. The generated cDNA needed to be stored at −80 °C. For better research, we conducted qRT-PCR using SYBR Green in our experiments and monitored it using the ABI7500 real-time PCR system. Gene expression analysis of the genes, including the NAC gene in A. sativa, was carried out using qRT-PCR with DNA melting curve analysis. *β-Actin* was used as an internal control and gene expression was normalized using the 2^−ΔΔCt^ method. Primers were designed according to NAC CDS with Primer Express 3.0 software. The primers used in qRT-PCR are listed in Table S2.

### 2.8. Plant Material and Treatments in A. sativa

(yanmaiCV), which were from the *A. sativa* Research Institute Heilongjiang Academy of Agricultural Sciences, were used in this study. In the greenhouse, we planted the seeds in a 3:1 (*w*/*w*) mixture of soil and sand, germinated them, and irrigated them with half-strength Hoagland solution once every 2 days [21]. The seedlings were grown in a night temperature of 16–18 °C, a day temperature of 22–24 °C, relative humidity of 65–80%, a 14/10 h photoperiod (daytime, 06:00–20:00), and a light intensity of 200–230 μmol m^−2^ s^−2^. After 4 weeks, the germinated seedlings were variously treated with 150 mM NaCl solution (salt), 150 mM NaHCO_3_ (saline alkali), cold treatment (8 °C), and freezing treatment (4 °C). The control and treated seedlings were harvested at 6 h, 12 h, 24 h, and 48 h after treatment. Samples were immediately frozen in liquid nitrogen and stored at −80 °C until used for RNA extraction.

### 2.9. Statistical Analysis

All the results were presented as a mean ± SD of at least three biological replicates. The qRT-PCR experiments were analyzed by one-way ANOVA followed by a post hoc least significant difference (LSD) test using the statistical software SPSS 20.0 (*p* < 0.05).

## 3. Results

### 3.1. Identification of NAC Genes in A. sativa

We identified 177 *NAC* genes from the oat genome. They are named *AsNAC1*~*AsNAC177* according to the sequence of their chromosomal positions. The length of the AsNACs proteins ranged from 362 bp to 3543 bp, and their relative molecular weights ranged from 22,781.6 to 222,299.2. The theoretical isoelectric points ranged from 4.42 to 10.19. Among them, 62 of the 177 genes had isoelectric points greater than 7 (within the basic range), one gene had isoelectric points equal to 7 (neutral), and the other 114 genes had isoelectric points less than 7 (within the acid range) (Table S1).

### 3.2. Genetic Analysis of NAC Genes in A. sativa

**Figure 1:**
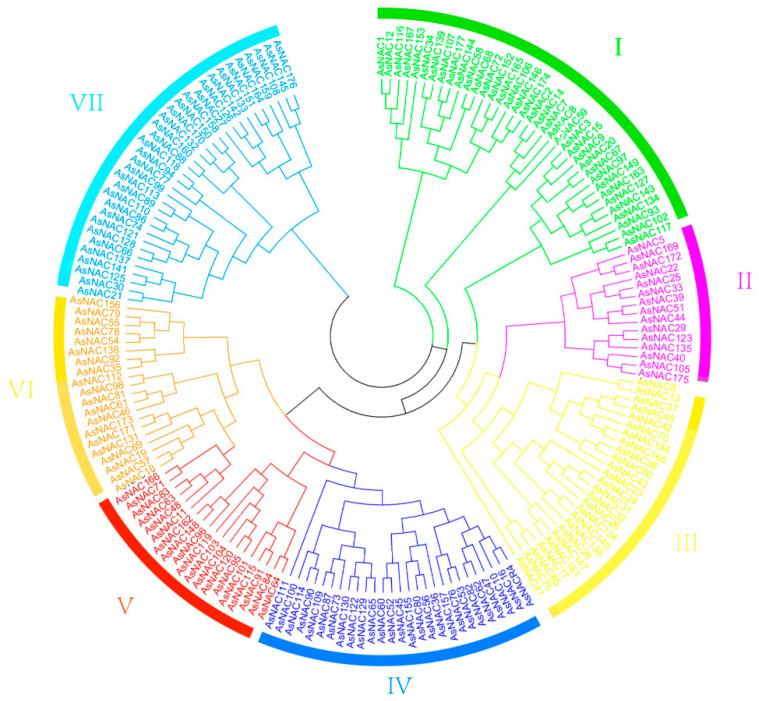
Systematic evolutionary relationships of NAC gene family (seven subfamilies were named subfamily Ⅰ~Ⅶ).

To study the classification of NAC gene family members, we constructed a phylogenetic tree of NAC protein sequences from *A. thaliana* (37), *O. sativa* (55), and *A. sativa* (100) (Figure S1). To further investigate the phylogenetic relationships of the NAC gene family in A. sativa, we constructed a phylogenetic tree (Figure 1) that divides the AsNACs genes into seven subfamilies (I–VII). Among them, subgroup II is the smallest with only 15 (8.5%) members; subgroup V has 19 (10.7%) members; subfamilies III, IV, VI, and VII have 26 (14.7%), 27 (15.3%), 20 (11.3%), and 33 (18.6%) members, respectively; and subgroup I is the largest with 37 (20.9%) members.

### 3.3. Motif Analysis, Gene Structures and Conserved Domains in A. sativa

**Figure 2:**
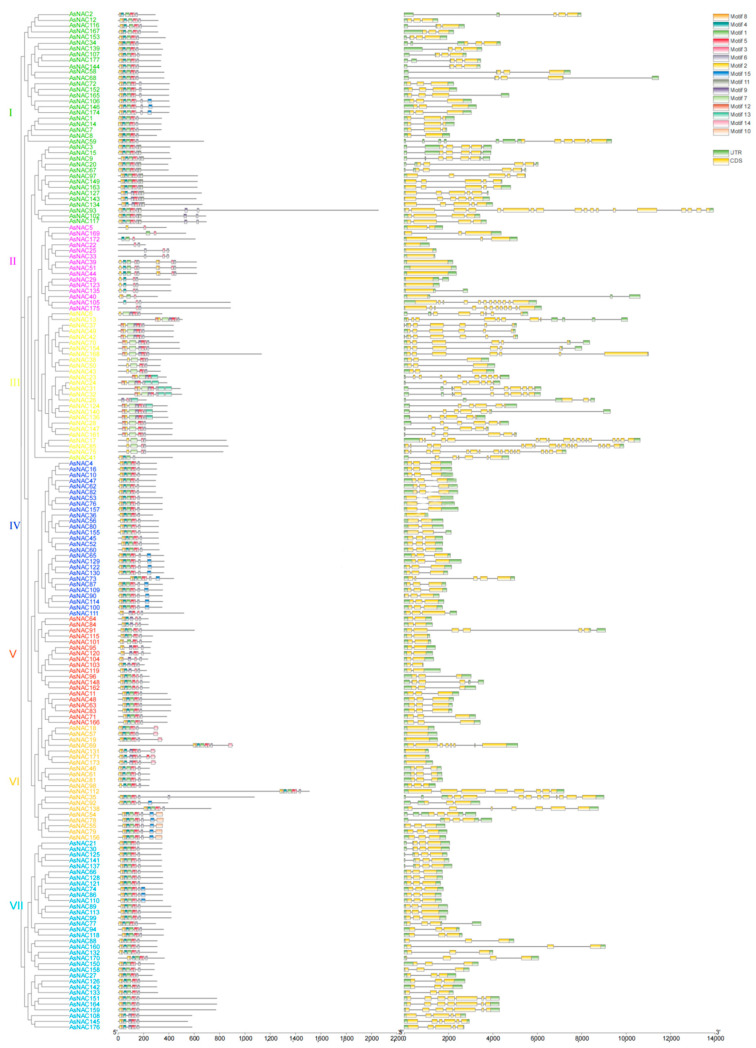
*NAC* gene family intron–exon and conserved domain analysis of the *NAC* protein. Phylogenetic tree in the first column of the figure; the motif composition and proportional motif length of the *NAC* protein in the second column; exon–intron structure of the third *NAC* gene in the third column (seven subfamilies were named subfamily Ⅰ~Ⅶ).

Fifteen motifs were identified in *AsNAC* gene family members, and the motifs have different types and quantities in the 177 genes (Figure 2). The class I, III, IV, V, and VII gene families contained eight conserved motifs. The class II gene family contains six conserved motifs. The class VI gene family contains 10 conserved motifs. Genes in the same subfamily has similar motif combinations, but some special motifs only occur in class I, such as Motif9, 11, and 15. We suggest that they may have special functions. In addition to the conserved motifs, the exon–intron pattern is also important for the different functions of NAC. The gene structure analysis showed that the NAC group consists of different numbers of exon–introns. The *AsNACs* genes contained 2~18 exons. There is little difference within a subfamily and a great difference across subfamilies in terms of the number of exons. For example, the number of exons in subfamilies II and VI was 1–3, and that in subfamilies III was 7–17. This suggests that different *AsNACs* subfamilies may have different functions.

In this study, we found 14 genes without an intron, accounting for 7.9%. The other genes have introns, accounting for 92.1%. The results indicate that most genes of the NAC gene family in oats have introns.

The NAC protein motif contains two incompletely repeated conservative domains. The *NAC* domain contains two extremely conserved arginine (R) residues and two highly conserved cysteines (C). In addition, it also contains other conservative amino acid residues, such as phenylalanine (F), serine (S), and aspartic acid (D).

### 3.4. Gene Duplication of NAC Genes

**Figure 3:**
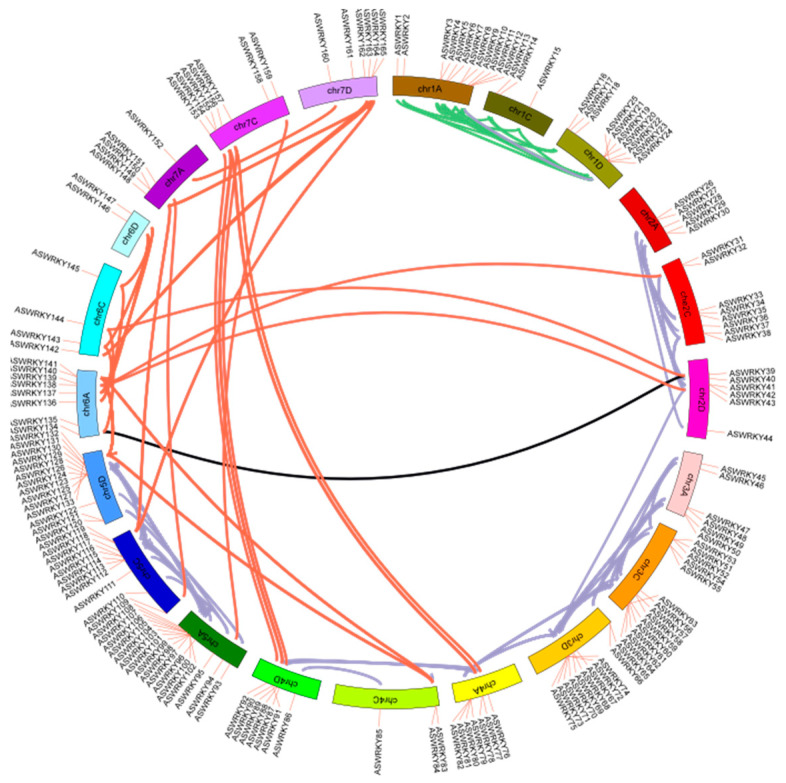
Analysis of chromosome location and duplication events in *A. sativa*. Genes of different subfamilies with gene duplication relationships are connected by lines in different colors.

The AsNACs genes are unequally distributed across the chromosomes. A total of 178 gene duplication events were detected in the NAC genes of the *A. sativa*. We constructed a chromosomal map showing the location of the NAC genes on each chromosome, and 177 *AsNACs* were located on 21 chromosomes in *A. sativa* (Figure 3). Among them, there are only 2 genes (at most 17) distributed on chromosome 2C. There are 136 pairs of fragment replications referring to 146 genes and 42 pairs of tandem replications referring to 95 genes. We observed that there are at most 69 homologous gene pairs in group D, 57 homologous gene pairs in group A, and 58 homologous gene pairs in group C in fragment replications. There are at most five genes distributed on chromosome 1. Genes are distributed on chromosome 2 (AsNAC6-11), chromosome 3 (AsNAC12-20), chromosome 4 (AsNAC21-25), chromosome 5 (AsNAC26-27), chromosome 6 (AsNAC28-36), chromosome 7 (AsNAC37-39), chromosome 8 (AsNAC40-48), chromosome 9 (AsNAC49-52), chromosome 10 (AsNAC53-64), chromosome 11 (AsNAC65-75), chromosome 12 (AsNAC76-84), chromosome 13 (AsNAC85-95), chromosome 14 (AsNAC98-108), chromosome 15 (AsNAC109-122), chromosome 16 (AsNAC123-130), chromosome 17 (AsNAC131-137), chromosome 18 (AsNAC138-144), chromosome 19 (AsNAC145-154), chromosome 20 (AsNAC155-160), and chromosome 21 (AsNAC161-177).

### 3.5. Synteny Analysis of the NAC Genes

**Figure 4:**
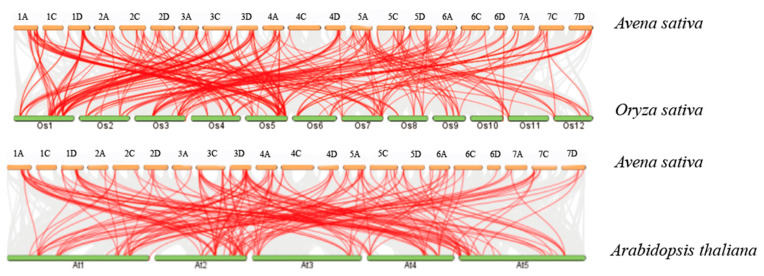
Synteny analysis of *WRKY* genes among *A. sativa*, *A. thaliala*, and *O. sativa*.

To further study the phylogenetic mechanism of the A. Sativa NAC family, we performed collinear analysis of NAC genes among *A. thaliala* and *O. sativas*. A NAC collinearity map among species was constructed for *A. thaliala* and *O. sativa* (Figure 4). *A. sativa* and *A. thaliala* have collinear gene pairs (198 pairs), of which *A. thaliala* homologous gene pairs are mainly distributed on 16 chromosomes. The homology of *A. thaliala*/*A. sativa* group D is the highest (77 genes). Group C is the lowest (47 genes). Group D has 74 homologous genes. *A. Sativa* and *O. sativa* have collinear gene pairs (264 pairs), of which *O. sativa* homologous gene pairs are mainly distributed on 18 chromosomes except chr2A, chr2C, and chr2D. The homology of *O. sativa*/*A. sativa* group A is the highest with 90 homologous genes. Group C is the lowest (86 genes). Group D has 88 homologous genes. The results showed that *A. sativa* has higher homology with rice from the same gramineae family than *A. thaliala* and rice.

### 3.6. Cis-Elements Analysis of NAC Genes

Promoter sequences were analyzed by using PlantCARE to identify cis-regulatory elements within 1500 bp upstream of the start codon. The promoter sequence predicted 165 genes containing hormone response elements, such as ABRE and P-box; 116 genes containing drought response elements (MBS), 73 genes containing low-temperature response elements (LTR), and 26 genes containing defense and stress response elements (TC rich repeats). A great number of elements related to growth were also found, such as G-box, ry element, and circadian (Figure S1). Therefore, it is speculated that the oat *NAC* gene may be widely involved in a variety of physiological and biochemical reactions during plant growth. AsNAC105 has part of a light-responsive element, a light-responsive element, a cis-acting regulatory element involved in light responsiveness, and a cis-acting regulatory element involved in MeJA-responsiveness. AsNAC60 has a light-responsive element, part of a light-responsive element, and a cis-acting regulatory element involved in light responsiveness. AsNAC35 has a light-responsive element, part of light-responsive element, a cis-acting regulatory element involved in light responsiveness, an auxin-responsive element, and a cis-acting element involved in abscisic acid responsiveness. AsNAC100 has a light-responsive element, part of a light-responsive element, a cis-acting regulatory element involved in light responsiveness, a cis-acting regulatory element essential for anaerobic induction, and part of a module for light response. AsNAC31 has a cis-acting regulatory element involved in light responsiveness, a binding site of AT-rich DNA binding proteins (ATBP-1), part of a light-responsive element, a light-responsive element, a cis-acting regulatory element involved in light responsiveness, and cis-acting regulatory element involved in MeJA-responsiveness. AsNAC50 has part of a light-responsive element, a light-responsive element, a gibberellin-responsive element, a binding site of AT-rich DNA binding proteins (ATBP-1), and an MYB binding site involved in light responsiveness.

### 3.7. Gene Regulatory Network Analysis of NAC Protein

**Figure 5:**
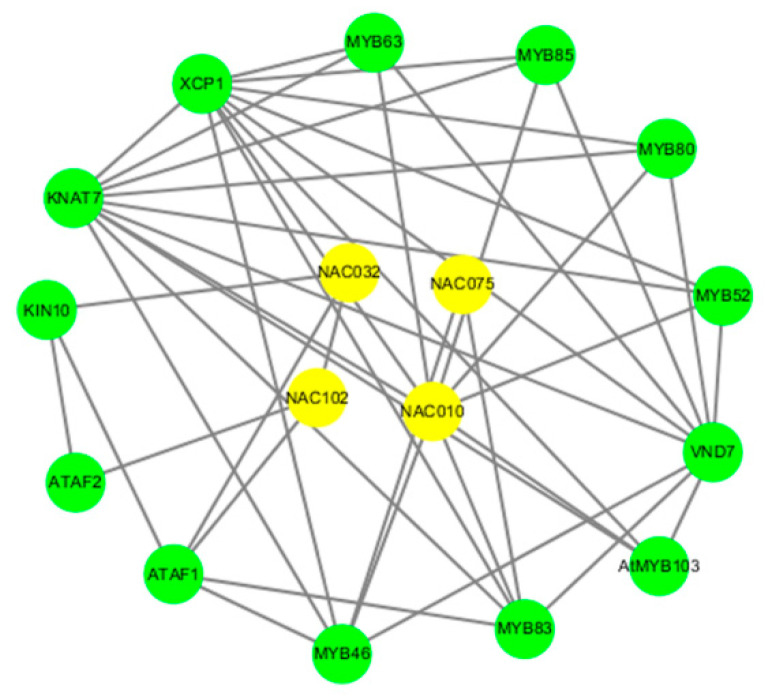
*A. sativa* NAC protein interaction network diagram. The yellow represents the NAC gene and the green represents the genes associated with NAC.

Furthermore, we found that the NAC proteins were completely localized to the NAC protein and other proteins in Arabidopsis. The interaction between the NAC genes and other proteins was predicted using the PAIR tool. The NAC protein is considered to be involved in different protein interactions (Figure 5), including transcription factor (MYB), protein kinase (KIN), semi fatty protease (XCP), transcription factor (KNAT), and apical meristematic protein (VND). In addition, we found that some NAC proteins interact, such as NAC32/NAC102, NAC102/ATAF1, NAC10/MYB, and NAC10/XCP1. NAC32 and KIN10 and ATAF1 have complex interaction relationships. NAC102 and ATAF2 and ATAF1 32 have complex interaction relationships. NAC10 and MYB52, MYB80, MYB85, MYB63, XCP1, KNAT7, MYB46, and MYB83 have complex interaction relationships.

### 3.8. Expression Patterns of NAC Genes in A. sativa under Abiotic Stress

**Figure 6:**
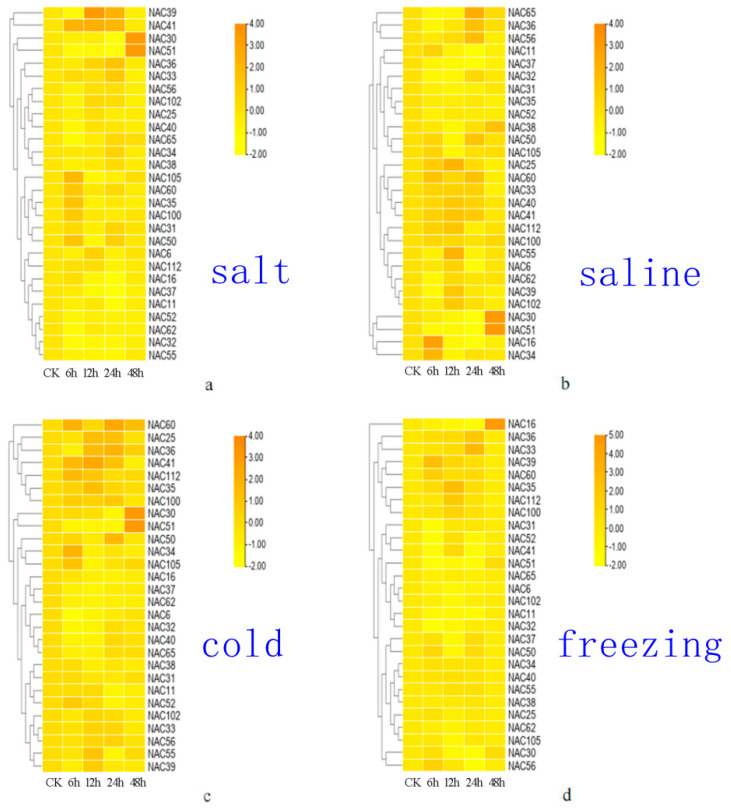
The expression patterns of *NAC* genes under four stresses. Expression analysis of NAC genes in different tissues in *A. sativa*, *A. thaliala* and *O. sativa*. (The first figure is the expression of salt stress, the second figure is the expression of saline stress, the third figure is the expression of cold stress and the fourth figure is the expression of freezing stress).

NAC transcription factors play critical roles in plant abiotic stress responses. In this study, the expression of *A. sativa* NAC genes in response to cold, freezing, salt, and saline alkaline abiotic stress were investigated using qRT-PCR. NAC1 transcription factors play critical roles in plant abiotic stress responses. The expression patterns of *AsNAC* genes under cold, freezing, salt, and saline alkaline abiotic stress at different times (CK, 6 h, 12 h, 24 h, 48 h) were analyzed using fluorescent quantitative PCR: Under cold stress, *AsNAC105*, *AsNAC60*, *AsNAC35*, *AsNAC100*, *AsNAC31*, and *AsNAC50* were down-regulated except for 6 h; and *AsNAC30* and *AsNAC51* were significantly up-regulated at 48 h (Figure 6a). Under freezing stress, *AsNAC16* and *AsNAC34* were down-regulated except for 6 h, while *AsNAC30* and *AsNACA51* were significantly up-regulated at 48 h (Figure 6b). We found that *AsNAC30* and *AsNACA51* were up-regulated under both stress conditions, and the difference multiple at 24 h was the highest. Under salt stress, *AsNAC60*, *AsNAC25*, *AsNAC36*, *AsNACA41*, *AsNAC112*, *AsNACA35*, and *AsNAC100* were up-regulated at 1 h, 6 h, and 12 h (Figure 6c). *AsNAC34* and *AsNAC105* were up-regulated only at 1 h, while *AsNAC30* and *AsNACA51* were significantly up-regulated at 48 h. Under saline and alkali stress, *AsNAC36* and *AsNAC33* were up-regulated at 24 h, *AsNACA16* was significantly up-regulated at 24 h, and *AsNAC11* and *AsNAC51* were down-regulated at 6 h, 12 h, and 24 h (Figure 6d).

## 4. Discussion

This study identified 177 AsNACs genes in the oat genome, which were classified into seven subgroups (I–VII), similar to the NAC gene classification of rice and soybean [12,22]. Our findings indicate that class I has the largest number of members while class V has the least. This is in contrast to cotton and cucumber. Class I has the largest number of members in cotton and class III has the smalleset. Class V has the most members in cucumber while class III has the least [23]. The study also found that subfamilies vary in proportion and specificity across different species.

NAC transcription factors have been identified in various plants, including 105 in Arabidopsis, 151 in rice, 168 in wheat, 147 in millet, and 152 in soybeans [1,12,13,24,25]. The phenomenon of multiple gene copies within a genome can be attributed to extensive replication and diversification during the process of evolution [26]. Our study revealed that the AsNAC fragment underwent more replication events through non-tandem duplication, with 136 pairs of fragment replication and 46 pairs of tandem replication. In Setaria italica, there were five pairs of fragment replication and only one pair of tandem replication [27]. A study found that fragment replication in Panicum miliaceum is greater than tandem replication, with 84 pairs of fragment replication and only 5 pairs of tandem replication. Similarly, non-Gramineous peanut (Leguminosae) has 116 fragment replications compared to only 1 pair of tandem replication [28]. However, Solanum melongena (Solanaceae) has more tandem replication than fragment replication [29]. These findings suggest that angiosperms primarily use fragment replication. Additionally, this study revealed that there are 246 pairs in oats and rice, and 198 pairs in oats and Arabidopsis in the whole genome collinearity analysis. This suggests that NAC transcription factors replicate more in closely related species during evolution.

The intron number of AsNACs genes ranged from 1–17, which is similar to rice, millet, and maize. Specifically, the intron number of rice, millet, and maize ranges from 1–16 [30], 0–14 [31], 0–14 [32], respectively. However, intron numbers are different to those of soybeans (1–7) [21], cassava (0–5) [33], and bananas (0–6) [34]. The results showed that the intron range of Gramineous crops is greater than that of other crops, which proves that the Gramineous NAC gene has high diversity in the gene structure.

Based on our analysis of the protein interaction network diagram, it appears that there may be an interaction between AsNAC16 and AsNAC53. This is supported by the fact that their homologous genes, OsNAC5 and OsNAC6, have been shown to exhibit higher tolerance to low temperature and salt stress than wild-type, and are induced by an ABA signal [35]. In our study, we discovered that AsNAC16 and AsNAC53 contain hormone response element ABA and are up-regulated under cold stress and saline alkali stress in qRT-PCR. Based on these findings, we suggest that AsNAC16 and AsNAC53 may function as transcription factors in ABA-dependent signaling pathways in response to abiotic stress.

Our study revealed that AsNAC60 contains a MYB cis-element binding site and has the potential to interact with MYB, as shown in our protein network interaction diagram. The homologous gene *TANAC071*-A of *AsNAC60* has been confirmed to activate the binding of the MYB transcription factor to TaMYBL1 by inserting two MYB elements, thus improving the water use efficiency of plants and increasing the expression of stress response genes to enhance the drought tolerance of overexpressed *TANAC071*-A plants [36]. AsNAC60 was found to be up-regulated in response to cold stress and significantly up-regulated in response to salt stress. This study demonstrated that the NAC gene has the ability to bind to promoter elements and up-regulate certain stress genes, thereby altering the abiotic stress tolerance of plants.

## 5. Conclusions

This study identified 177 NAC genes from the oat genome, categorized into 7 subfamilies. Genes within the same subgroup share similar gene structures and motifs. Phylogenetic analysis showed that AsNACs proteins can be divided into seven subfamilies (I–VII), and that proteins in the same subfamily have similar protein motifs. Gene structure analysis found that NAC introns ranged from 1 to 17. Cis-element analysis of promoters indicated that the gene family may have stress-related elements and growth regulation elements. qRT-PCR analysis revealed that 9 genes were significantly expressed under cold treatment, while 10 genes were significantly expressed under drought treatment. Additionally, seven genes were up-regulated under salt stress, whereas only five genes were up-regulated under saline alkali stress. This study found that NAC genes exhibited a higher response to freezing stress and salt stress, as compared to cold stress and saline alkali stress. Additionally, the genes were found to respond to ABA and GA. These findings provide a theoretical basis for further research on the function of the NAC gene family.

## Data Availability

Not applicable.

## Supplementary Materials

The following supporting information can be downloaded at: https://www.mdpi.com/article/10.3390/genes14061186/s1, Figure S1: Analysis of the promoter elements of the *NAC* gene family in *A. sativa*; Table S1: List of *NAC* genes information identified in *A. sativa*. Table S2: List of primers used in qRT-PCR.
