# Supplementary figures and images for: Genome-Wide Identification of NAC Gene Family and Expression Analysis under Abiotic Stresses in Avena sativa
Source: Genes (Basel). 2023 May 29;14(6):1186. doi: 10.3390/genes14061186 (PMC10298501; doi:10.3390/genes14061186)

## Figure S1

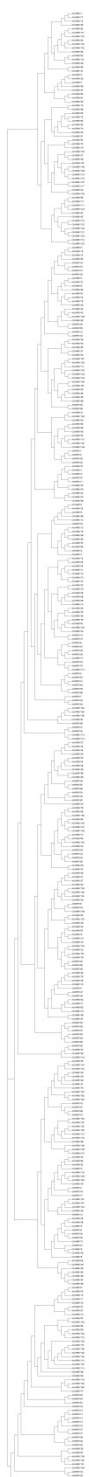

## Figure S2

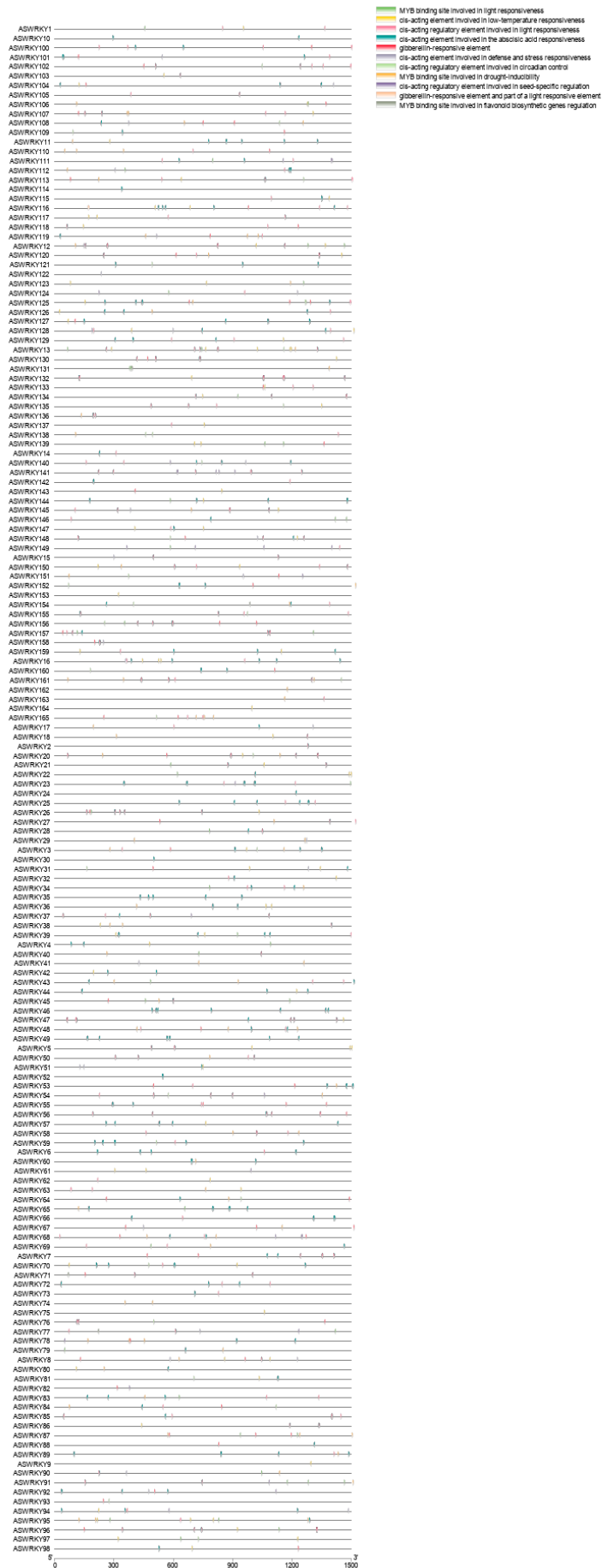

Supplement: Supplementary file 1 [file genes-14-01186-s001.zip › FigureS1-S2.pdf]
